# Supplementary material for: Complexome profiling of the Chlamydomonas psb28 mutant reveals TEF5 as an early PSII assembly factor
Source: Plant Cell. 2025 Mar 17;37(6):koaf055. doi: 10.1093/plcell/koaf055 (PMC12164590; doi:10.1093/plcell/koaf055)
Supplement: koaf055_Supplementary_Data [file koaf055_supplementary_data.zip › psb28_interactive.zip!/plots/intensity/qualityPlots/peptides/_Cre13.g584250.t1.2.html]

### Cre13.g584250.t1.2

Trivial name:   
SeqCoverage [median]: 17.70  
Mass: 31.22 kDa  
  
 

| Sequence | MissedCleavage | Mass [Da] | wtIntensity | muIntensity | Score |
| --- | --- | --- | --- | --- | --- |
| AVAQDDYLSER | 0 | 1265.59 | 872.93 | 10209.69 | 101.11 |
| LEPVEDELDMYGRPK | 1 | 1789.86 | 3583.39 | 19736.86 | 78.49 |
| LLAAYR | 0 | 705.42 | 0.00 | 3098.68 | 85.08 |
| LVDELTPK | 0 | 913.51 | 0.00 | 1409.62 | 67.03 |
| TYVDTTDTRPR | 1 | 1323.64 | 7263.63 | 22329.85 | 99.52 |
|  |  |  | 11720.0 | 56784.7 |  |
